# Effects of Two-Step Transamidation of Wheat Semolina on the Technological Properties of Gluten

**DOI:** 10.3390/foods5030049

**Published:** 2016-06-29

**Authors:** Salvatore Moscaritolo, Lucia Treppiccione, Antonio Ottombrino, Mauro Rossi

**Affiliations:** 1 Unit for Cereal Quality (CRA-QCE), Council for Agricultural Research and Economics Research, Roma 00189, Italy; salvatore.moscaritolo@crea.gov.it; 2 Institute of Food Sciences, CNR, Avellino 83100, Italy; luciatrep@gmail.com (L.T.); aottombrino@isa.cnr.it (A.O.)

**Keywords:** celiac disease, transamidation, wheat semolina

## Abstract

Celiac disease (CD) is an immune-mediated disorder caused by the ingestion of wheat gluten. A lifelong, gluten-free diet is required to alleviate symptoms and to normalize the intestinal mucosa. We previously found that transamidation reaction by microbial transglutaminase (mTG) was effective in down-regulating the gliadin-specific immune response in CD patients. In this study, the two-step transamidation protocol was adopted to treat commercial wheat semolina on a pilot scale. The effectiveness of the enzymatic reaction was tested by means of consolidated biochemical and immunological methods on isolated prolamins. We found that water-insoluble gliadin and glutenin yields decreased in wheat semolina to 5.9% ± 0.3% and 11.6% ± 0.1%, respectively, after a two-step transamidation reaction. Using DQ8 transgenic mice as a model of gluten sensitivity, we observed a dramatic reduction in IFN-γ production in spleen cells challenged in vitro with the residual insoluble gliadin from transamidated semolina (*N* = 6; median values: 850 vs. 102; control vs. transamidated semolina, *p* < 0.05). The technological properties of treated wheat semolina were then tested by manufacturing classical pasta (spaghetti). Notably, the spaghetti manufactured with transamidated semolina had only minor changes in its features before and after cooking. In conclusion, the two-step transamidation reaction modified the immunogenic epitopes of gliadins also on a pilot-scale level without influencing the main technological properties of semolina. Our data shed further light on a detoxification strategy alternative to the current gluten-free diet and may have important implications for the management of CD patients.

## 1. Introduction

Celiac disease (CD) is an immune-mediated disorder caused in genetically susceptible individuals by the ingestion of wheat gluten and related prolamins present in barley and rye [1]. CD affects approximately 1% of the general population in developed and developing countries, with an increasing prevalence reported in Europe and the USA [2,3]. Currently, a lifelong, gluten-free (GF) diet is required to alleviate the symptoms of CD and to normalize the antibodies in the intestinal mucosa [3]. However, dietary compliance is poor, necessitating the development of alternative technological strategies to treat CD. Furthermore, gluten plays a key role in establishing the unique rheological properties and baking quality of wheat, which are partially restored in GF products. Moreover, to improve palatability, many GF products are manufactured with purified wheat starch, which invariably contains residual gluten. Gluten proteins are divided into two fractions according to their solubility in alcohol-water solutions: gliadins (soluble) and glutenins (insoluble). Both components contain high levels of glutamine (30%–35%) and proline (10%–15%) residues and very few negatively charged amino acids. These proteins undergo a process of selective deamidation in the small intestine of CD patients, during which specific glutamine residues are converted to glutamic acid by tissue transglutaminase (tTG) [4]. The presence of many proline residues in these proteins, which are resistant to digestive enzymes, ensures that many immunostimulatory epitopes survive digestion [5]. Notably, a previous study found that gliadin can be cleaved by bacterial prolyl endopeptidases (PEPs) into short peptides that then lose their activity [6]. Accordingly, PEPs have been evaluated as a technological tool for the preparation of detoxified gluten. One study reported that a 60-day diet of baked goods made from PEP-hydrolyzed wheat flour was not toxic to CD patients [7]. To improve the preservation of the gluten structure we tested a different enzymatic approach using the transamidation activity of food-grade microbial transglutaminase (mTG), a transamidase of the endo-γ-glutamine:ε-lysine transferase type [8]. Unlike tTG, mTG is a calcium-independent, low molecular weight protein, which has several advantages for food industrial applications [9]. This enzyme is commercially available as a dough improver that adds stability and elasticity to dough [10]. Importantly, the covalent attachment of amino acids by enzymatic procedures is also a generally accepted means of improving the nutritional quality and functional properties of food proteins. Previous studies have shown that the presence of the isopeptide linkages in gliadins does not impair their digestibility [11], indicating that this treatment is safe. The final catabolic step in gluten transamidation occurs largely in the kidneys, where ε-(γ-glutamyl)-lysine provides a substrate for γ-glutamylamine cyclotransferase [12]. Importantly, we found that the transamidation of gliadin following the treatment of wheat flour with mTG and lysine methyl ester caused a dramatic down-regulation in IFN-γ production in vitro in the intestinal T cells of CD patients [13]. Furthermore, we demonstrated that wheat flour, following transamidation using a new “two-step” procedure with lysine ethyl ester, was selectively associated with positive changes in the phenotype of the antigen-specific immune response in models of gluten sensitivity [14]. The present work investigated the reaction products of wheat semolina following transamidation using the two-step procedure on a pilot scale.

## 2. Materials and Methods

### 2.1. Quality Characteristics of Durum Wheat Semolina

The chemical and technological characterizations of semolina were performed by standard analyses: protein content (ICC 105/2 Kieldhal), gluten content % (ICC 137/1; 155; 158), yellow index (Minolta Chromameter CR-300, CEN standard method 15465), alveographic test (ICC 121), Braabender Farinograph (ICC 115/1). A commercial durum wheat was used for testing. Semolina was obtained by a pilot milling plant (Buhler MLU 202, Uzwil, Switzerland). Data are referred as mean of repeated analyses and differences between replicates were included within the specific ranges of each method.

### 2.2. Transamidation Reaction of Durum Wheat Semolina

Food-grade microbial transglutaminase (mTG) was from Ajinomoto Foods (Hamburg, Germany; ACTIVA^®^WM; 81–135 U/g); lysine ethyl ester (K-C_2_H_5_) was from NutraBio (NutraBio.com, Middlesex, NJ, USA). Semolina was suspended in two volumes of water containing 8 U/g mTG and 20 mM K-C_2_H_5_. Incubation was performed in a reactor plant Micro MFCS (BBraun AG, Melsungen, Germany) with a nominal capacity of 16 liters. The reactor plant was preliminary sterilized, then the temperature was decreased at 30 °C. The first step was conducted for 2 h at 30 °C and the suspension was recovered by centrifugation (1000× *g*, 10 min). After an extensive washing of the reactor with tap water, a second enzyme step was conducted for 3 h at 30 °C with fresh enzyme and K-C_2_H_5_ at the same concentrations. The suspension was finally centrifuged (15,000× *g*, 10 min) and dough recovered.

### 2.3. Biochemical Analysis of Transamidation of Wheat Semolina

A sample of 20 mL semolina suspension was centrifuged at 3000× *g* for 10 min. The residual gliadin and glutenin fractions were extracted from the protein pellet using a modified Osborne procedure [15]. Proteins content was assessed by Bradford analysis [16].

### 2.4. Immunological Analysis of Transamidation of Wheat Semolina

Transgenic mice expressing the HLA-DQ8 molecule in the absence of endogenous mouse class II genes [17] were reared for several generations on a GF diet (Altromin-MT-mod, Rieper SpA, Bolzano, Italy) in pathogen-free conditions at our animal facility (accreditation n. 164/99-A). All procedures met the guidelines of the Italian Ministry of Health. Six-week-old mice were primed by intraperitoneal injection with gliadins (300 μg) emulsified in Freund’s complete adjuvant (Sigma) (day 0). Boosters containing the same amount of antigen in incomplete Freund’s adjuvant were injected on days 7 and 14. Mice were sacrificed on day 21 to recover their spleens. Spleens were passed through a stainless steel wire mesh to dissociate cells. Erythrocytes were removed by treating the cell suspensions with a Tris-buffered ammonium chloride solution. For each sample, 5 × 10^5^ cells were incubated in 0.2 mL culture medium in 96-well flat bottom plates at 37 °C for 96 h in the presence of gliadins (200 μg/mL). After 72 h, the supernatants were collected and analysed for IFN-γ protein levels by in-house sandwich ELISA.

### 2.5. Pasta Manufacturing Procedure

Semolina was used to produce pasta samples by a pilot plant (Namad—Rome, Italy). The transamidated wet dough, showing an hydration index of 30%, was homogenized for 5 min at room temperature in a kneader pre-mixer and then transferred to the mixing chamber under vacuum. In the next step, transamidated wet dough was pressed up to 100 bar through a screw (300 mm long and 45 mm diameter) at 30 °C. The dough was shaped into spaghetti (size ∅ = 1.30 mm) using a bronze extruder. Then pasta was dried by a pilot plant drying device (Afrem—Clextral sas, Firminy, France) adopting the following temperature (°C)/relative humidity (rh) schedule: 77 °C/ 85% rh, 3 h; 70 °C/ 77% rh, 3 h; lowering from 70 °C to 35 °C/ 70% rh, 1 h; 35 °C/ 65% rh, 20 h; dried pasta was finally stored at room temperature under controlled atmosphere. Pasta cooking quality was evaluated by sensory analysis according to D’Egidio et al. [18]. Results related to the quality aspects were expressed as mean values of three determinations.

### 2.6. Statistical Evaluation

Statistical significance was determined by the Kruskal-Wallis test and Dunn’s post-hoc test analysis using GraphPad PRISM 4.0 software (GraphPad Software, Inc., La Jolla, CA, USA). A *p*-value of 0.05 or less was considered to be significant.

## 3. Results

### 3.1. Qualitative Features of Semolina and Dried Pasta

The chemical and rheological characteristics were evaluated by classical methods: the protein content of semolina was 12.4% ± 0.1% (d.m.) with a gluten content of 10.3% ± 0.1% (d.m. basis); the yellow index (b) was 17.5 ± 0.1 and 15.7 ± 0.2 for semolina and dried pasta, respectively; and the rheological characteristics gave results of an alveographic test of W 210 (10^−4^ Joule) and p/L 4.5 and a farinograph braabender with an absorption of 55% and stability 5.0 min.

### 3.2. Pilot-Scale Production of Transamidated Semolina

We empirically determined that the best reactor performance could be achieved by enzymatically treating a maximum of 6.0 kg of durum wheat semolina in a final volume of 13.5 L. Accordingly, semolina was slowly suspended in 10.0 L of 20 mM K-C_2_H_5_ water solution and the suspension was transferred into the reactor. ACTIVA^®^WM was gently added under stirring conditions. Subsequently, the mixing speed was increased and we found that 450 rpm was needed to obtain a uniform distribution of the enzyme. After centrifugation the pellet was suspended in 20 mM K-C_2_H_5_ (12.0 L final volume) to perform the second step. By adopting this approach, we obtained a yield of 9.6 kg of transamidated dough.

### 3.3. Analysis of Transamidated Prolamins

**Figure 1:**
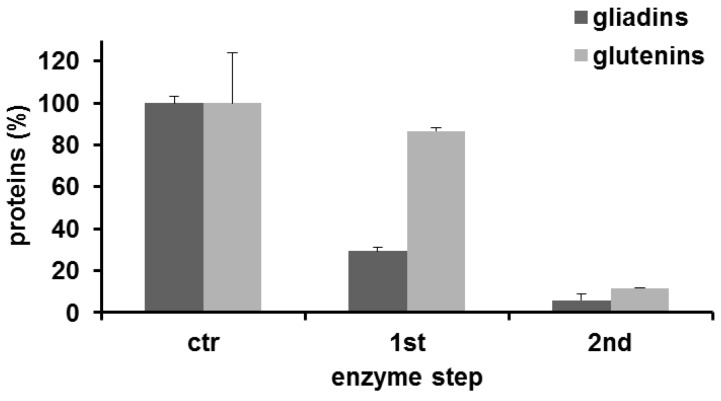
Assessment of the residual gliadin and glutenin protein fractions following the transamidation reaction, purified according to the modified Osborne procedure. Each bar represents values (means ± SD) calculated as the percentage of control (untreated semolina) of triplicate experiments.

**Figure 2:**
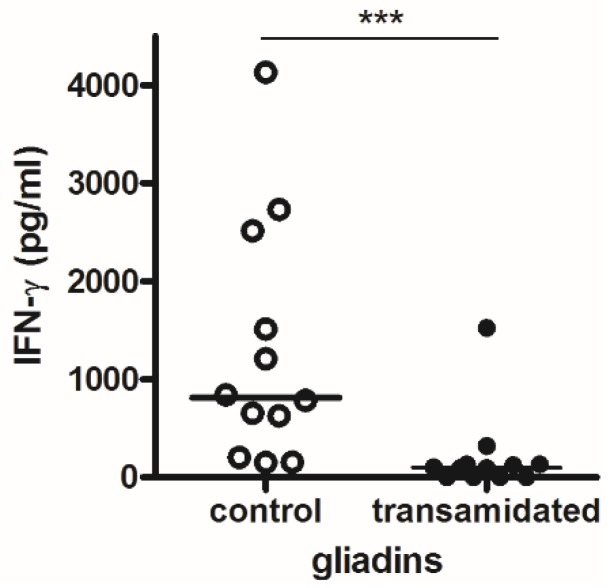
Antigen-specific IFN-γ expression assessed after culturing spleen cells from immunized mice for 72 h (*N* = 12). Each dot represents values (pg/mL) from a single mouse calculated as the difference between the means of triplicate cultures containing antigen and triplicate cultures with medium alone. Control is the cytokine response to native gliadin. *** *p* < 0.001.

The production of isopeptide bonds from the catalytic activity of mTG dramatically decreased the gliadin yield to 29.3% ± 1.9% and 5.9% ± 0.3% after the second step (mean ± SD; Figure 1). On the contrary, the glutenins yield was fairly affected after the first enzyme step (86.6% ± 1.6%), and it decreasing to 11.6% ± 0.1% after the second step. Next, we focused on the immunological effects of gliadins extracted from semolina following a two-step transamidation process. To determine possible modifications in the T cell–mediated response, we used HLA-DQ8 transgenic mice, which only express the human MHC class II molecule that has been linked to CD [17]. Following immunization with gliadins, spleen cells were recovered and stimulated in vitro with different gliadin preparations. The immune response was analyzed by evaluating the IFN-γ expression. Results shown in Figure 2 indicated that spleen cells from immunized mice induced significant cytokine protein levels after a 72 h culture when stimulated with native gliadin. Notably, when gliadin-specific spleen cells were stimulated with residual insoluble gliadin isolated from semolina subjected to a two-step transamidation, the production of IFN-γ was dramatically blocked.

### 3.4. The Rheological Properties of Transamidated Semolina

**Figure 3:**
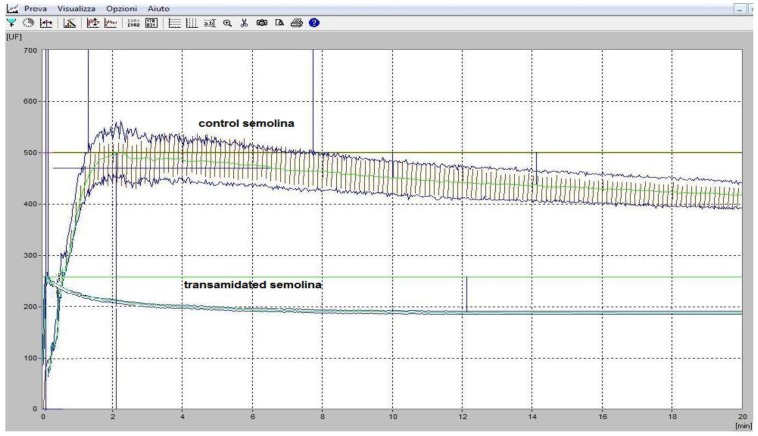
Farinograph profile of control and transamidated semolina.

After enzyme processing, transamidated wet dough showed a hydration index of 50% with a protein content that decreased to 7.2% (d.m.). As shown in Figure 3, we found a complete loss of classical rheological parameters in the transamidated product. Nevertheless, by performing a preventive drying treatment, which was needed to lower hydration to 30%, wet dough could be normally processed for pasta manufacturing. 

### 3.5. The Technological Properties of Transamidated Semolina

After cooking, the water uptake in transamidated pasta was lower than in the control sample (200 g and 280 g wet weight, respectively, Table 1). Notably, the main cooking features were found unchanged in the spaghetti manufactured with transamidated semolina in comparison with commercial pasta (Table 1).

## 4. Discussion

This study showed that the pilot-scale two-step transamidation of wheat semolina was able to completely block the immune recognition of wheat gliadins in vivo. Importantly, this treatment did not hamper the main technological properties of gluten, as good quality dried pasta was produced.

CD is an inflammatory autoimmune disease of the small intestine [1] that affects genetically susceptible individuals. A major hallmark of CD is inappropriate intestinal T cell activation triggered by peptides from wheat gliadins and glutenins, and by related prolamins from barley and rye. To date, several gliadin epitopes that elicit a T cell response have been identified, and most of these were recognized following tTG-mediated deamidation of specific glutamine residues [4,19]. Based on these findings, we previously reported that transamidation of gliadin by mTG suppressed the immune response of intestinal T cell lines from CD patients [13]. tTG and mTG have very different structures; furthermore, mTG has developed a calcium-independent catalytic mechanism [20]. However, we showed similar site-specificity for their catalytic activity [13]. It must be underlined that this detoxification strategy does not require a complete knowledge of all the toxic sequences present in gluten. This is an important clue considering that gluten is characterized by an intrinsic complexity, which contains hundreds of related proteins present either as monomers or linked by inter-chain disulphide bonds [15]. Hydrated gliadins are the proteins that primarily contribute to dough viscosity and extensibility. Hydrated glutenins are both cohesive and elastic and are responsible for dough strength and elasticity. In this study, we confirmed that the solubility of both gliadins and glutenins was drastically changed following mTG treatment of wheat semolina in the presence of K-C_2_H_5_: more than 94% gliadins and 88% glutenins, respectively, became soluble in water as a consequence of isopeptide bond formation. Most importantly, wheat semolina transamidation mediated by mTG modified the immunogenicity of gliadins in vitro, which is instrumental for generating celiacogenic epitopes. Furthermore, the transamidated forms were characterized by a complete loss of immune cross-reactivity toward anti-native gliadin antibodies [21]. In particular, R5-ELISA, a commercially available immunoassay used to detect residual gluten in GF foods, indicated that the transamidation of wheat flour in the presence of K-C_2_H_5_ causes effective epitope masking to occur in gliadin components [20]. In a first dietary intervention study, the reintroduction of gluten after a one-step treatment reduced the number of clinical relapses in challenged CD patients [22]. Nevertheless, the one-step reaction was not found sufficient in eradicating the gluten activity in all examined CD patients. Whether the herein-reported upgraded reaction is effective in fully blocking relapses in treated CD patients is currently under investigation. On the basis of the above reported data, dough recovered from the durum wheat semolina suspension after the two-step reaction contained most of the gluten that was transamidated. Nevertheless, we produced pasta with no changes in its technological and organoleptic parameters. It is known that the physical properties of a dough arise from interactions between gluten proteins, particularly the disulphide-bonded glutenin macropolymer [23]. Our data suggested that transamidated gluten can still hold most of these features by considering that disulphide bonds are not involved.

## 5. Conclusions

We demonstrated that the detoxification protocol of durum wheat semolina on a pilot scale, based on the use of food-grade mTG and lysine ethyl ester, largely produced transamidated water-soluble gliadins and glutenins; furthermore, it was selectively associated with a full reduction of the gliadin-specific immune response in wheat semolina. Notably, the enzyme treatment preserved the main technological properties of gluten. Our data may have important implications for the management of CD patients by shedding further light on an innovative enzymatic strategy as an alternative to the gluten-free diet.

## Figures and Tables

**Table 1:** Structural characteristics of pasta after cooking.

Parameters	Control Pasta	Experimental Pasta
Ø (mm) fresh pasta after extrusion	nd	1.6 (±0.0)
Ø (mm) raw dried pasta	1.3 (±0.0)	1.3 (±0.0)
cooking time until disappearance of the nucleus (min)	7	6
pasta weight after cooking (t 0’) (g)	280 ± 0.6	200 ± 0.5
pasta weight after cooking (t 9’) (g)	270 ± 0.3	193 ± 0.2
springiness	70 (±5)	70 (±3)
firmness	75 (±5)	75 (±5)
stickiness (t 9′)	70 (±5)	70 (±5)
taste (t 9′)	70 (±5)	90 (±3)
appearance (t 9′)	70 (±5)	80 (±3)
flavor (t 9′)	70 (±5)	75 (±5)

## Abbreviations

The following abbreviations are used in this manuscript:

CD	coeliac disease
GF	gluten free
K-C_2_H_5_	lysine ethyl ester
PEPs	prolyl endopeptidases
mTG	microbial transglutaminase
tTG	tissue transglutaminase
